# Maintenance of phenotypic diversity within a set of virulence encoding genes of the malaria parasite *Plasmodium falciparum*

**DOI:** 10.1098/rsif.2015.0848

**Published:** 2015-12-06

**Authors:** Thomas Holding, Mario Recker

**Affiliations:** Centre for Mathematics and the Environment, University of Exeter, Penryn Campus, Penryn TR10 9EZ, UK

**Keywords:** *Plasmodium falciparum*, *var* genes, phenotypic diversity, evolutionary trade-off, mathematical modelling

## Abstract

Infection by the human malaria parasite *Plasmodium falciparum* results in a broad spectrum of clinical outcomes, ranging from severe and potentially life-threatening malaria to asymptomatic carriage. In a process of naturally acquired immunity, individuals living in malaria-endemic regions build up a level of clinical protection, which attenuates infection severity in an exposure-dependent manner. Underlying this shift in the immunoepidemiology as well as the observed range in malaria pathogenesis is the *var* multigene family and the phenotypic diversity embedded within. The *var* gene-encoded surface proteins *Plasmodium falciparum* erythrocyte membrane protein 1 mediate variant-specific binding of infected red blood cells to a diverse set of host receptors that has been linked to specific disease manifestations, including cerebral and pregnancy-associated malaria. Here, we show that cross-reactive immune responses, which minimize the within-host benefit of each additionally expressed gene during infection, can cause selection for maximum phenotypic diversity at the genome level. We further show that differential functional constraints on protein diversification stably maintain uneven ratios between phenotypic groups, in line with empirical observation. Our results thus suggest that the maintenance of phenotypic diversity within *P. falciparum* is driven by an evolutionary trade-off that optimizes between within-host parasite fitness and between-host selection pressure.

## 1. Introduction

*Plasmodium falciparum* is the most virulent human malaria parasite. A key virulence determinant is the ability of infected red blood cells to bind to a variety of host receptors, causing sequestration in the deep vasculature and obstruction of blood flow in vital organs. Cytoadhesion is mediated by the highly polymorphic *Plasmodium falciparum* erythrocyte membrane protein 1 (PfEMP1), expressed on the surface of infected red cells and encoded by the *var* multigene family [1–4]. These proteins are also prominent targets for host immune responses, and each parasite genome contains a different repertoire of ≈60 *var* genes, of which only one gene is actively transcribed at a time. In a process known as clonal antigenic variation, parasites switch expression between individual *var* genes in a mutually exclusive fashion, causing new variants to appear over the course of an infection [5,6].

Different PfEMP1 variants bind to different host receptors [3] and can lead to parasite sequestration in different host tissues, such as the brain or placenta [7]. Probably, the best known example of this is the involvement of *var2csa* in pregnancy-associated malaria, which can be found in every *var* gene repertoire sequenced to date and whose protein product (*VAR2CSA*) binds exclusively to placental CSA, leading to severe complications during pregnancy [8]. Therefore, *in vivo* antigenic variation not only aids immune evasion, but can also evoke temporal changes in the phenotypic profile of the parasite population during infection, with important consequences for infection outcome. Note, for the purpose of this work, we refer to phenotype or phenotypic group, as a subset of *var* genes with similar binding characteristics.

Although hosts living in malaria-endemic areas may never attain a state of sterile immunity, they acquire a broad spectrum of PfEMP1-specific antibodies with repeated exposure [9–12]. This has been shown to attenuate infection severity, such that severe and fatal malaria is usually confined to the youngest age groups, whereas older individuals only rarely experience clinical malaria episodes [13]. Underlying this transition in the immunoepidemiology of *P. falciparum* malaria appears to be a qualitative change in *var* gene expression during infection mediated by the host's immune repertoire [14,15], indicating an exposure-mediated link between acquired immunity, phenotype selection and malaria pathology.

Despite their enormous sequence diversity, *var* genes can be grouped according upstream promoter sequence (Ups) or chromosomal location, with a remarkably conserved distribution of these groups within parasite repertoires [16,17]. This conserved partitioning appears to be facilitated by recombination being largely confined to within groups, meaning that groups may be evolving independently of one another [7,18]. Furthermore, these genetically defined groups seem to associate with binding phenotype [19], which suggests that *P. falciparum* parasites have not only evolved to maximize antigenic diversity, but also evolved a mechanism by which they maintain phenotypic diversity within their repertoires of antigen encoding and virulence-associated genes.

While the evolutionary forces that favour phenotypically diverse antigen repertoires remain largely unknown, the central role that PfEMP1 plays in acquired immunity and immune evasion makes selection by the immune system a likely candidate. Previous work exploring the effects of immune selection predominantly focused on between-host interactions and predicted that parasite populations will self-organize into antigenically discordant strains, thereby minimizing the detrimental effect of previous exposures on subsequent infections owing to cross-immunity [20]. The highly conserved partitioning of *var* gene repertoires, which has also been shown to restrict the number of unique antigen combinations and thus only poorly uses the available global pool of antigenic variants [21], is difficult to reconcile with this hypothesis, however.

More recently, we have postulated how evolutionary trade-offs at multiple ecological levels (within-host and within-populations) can explain observed distributions of *var* genes and gene domains with respect to their degree of sequence conservation [22]. Crucially, while this framework explored the evolution of gene and domain distributions within already partitioned repertoires, it did not address why parasites should encode multiple phenotypic groups, rather than evolving into separate and independently transmissible phenotypes.

Here, using an evolutionary framework, we show how a trade-off between within-host fitness and between-host selection pressure can robustly select for phenotypically diverse antigen repertoires. Furthermore, we show that functional constraints that limit the degree by which different phenotypes can diversify without losing binding functionality are sufficient to explain observed *var* gene repertoire structures in *P. falciparum*.

## 2. Methods

We developed a stochastic, individual-based model to investigate the evolutionary factors that select for phenotypically diverse antigen repertoires. We defined parasites strains by their antigenic repertoire, which itself comprised up to two phenotypic groups. That is, each repertoire was made up of antigenic variants of either phenotype *A* or phenotype *B*. We assumed that all strains have the same repertoire size, referred to as *r*, but could differ in the number of variants belonging to either group *A* or *B*. We refer to a particular repertoire structure as (*A* : *B*), indicating the ratio of antigens within the repertoire belonging to phenotype *A* or phenotype *B*. Note, repertoire structure can be considered as a collective term, referring to all strains with the same distribution of genes of type *A* and type *B*.

Owing to the high dimensionality of the potential antigenic space that can be generated simply through recombination of the global pool of antigenic variants, we investigated gene repertoire evolution by repeatedly simulating population infection histories with a fixed set of parasite strains. At the beginning of each simulation, we initialized the parasite population to contain exactly one member of every possible repertoire structure (i.e. one of each (*r* : 0), (*r* − 1 : 1), … , (0 : *r*)) and filled the repertoires by randomly sampling from the two global variant pools (of sizes *N*_A_ and *N*_B_). Each simulation was run until it reached a dynamic equilibrium (around 10 000 simulated days), at which point all but a small subset of strains are driven to extinction. We refer to those strains that are maintained within the population as ‘dominant’, irrespective of their population-level prevalence. That is, dominance is here defined as a purely qualitative measure referring to those strains that have competitively excluded all other strains.

For the majority of simulations, we restricted the genomic repertoire sizes to *r* = 9 and antigenic pool sizes to *N*_A_ = 13 and *N*_B_ = 13, which are all significantly smaller than the assumed diversity of *var* genes both within individual repertoires and at the population level. These numbers were chosen for computational reasons only (larger numbers take significantly longer to converge), however, and we note that our results were independent of repertoire and global variant pool sizes. Examples of larger repertoire and variant pool sizes can be found in electronic supplementary material, figure S1.

Hosts are modelled individually with the host population being kept constant at 10 000 and deaths being replaced by newborns. Host mortality was assumed to be age-dependent, given as2.1
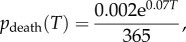
where *T* is the host age in years. We ignored maternal protection and further assumed that infections do not contribute to death. At the start of each simulation, hosts are initialized such that the population is demographically at equilibrium.

For the sake of simplicity, we kept the total rate of infection constant over time and instead assumed transmission to be solely frequency dependent, where the (daily) probability of a host getting infected with strain *i* depends only on the relative frequency of strain *i* in the population. The daily infection rate of strain *i* can then be given as2.2
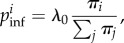
where *λ*_0_ = 0.033 is the daily force of infection and *π_i_* is the proportion of hosts currently infected with strain *i*.

Upon infection, it was assumed that the infecting strain will express its antigenic repertoire in a random order, where each variant contributes positively to infection length. Hosts acquire immunity in a variant-specific manner, which prevents the specific variant from contributing to future infections. In this fashion, hosts might acquire full immunity to a particular strain without necessarily having been exposed to it. Where cross-immunity is considered, not all variant genes will contribute equally to infection length, in which case variant-specific immunity is acquired probabilistically and in proportion to their contribution to infection length, *L*.

Within this framework, a strain's fitness solely refers to infection length and is therefore not a fixed quantity, but dependent on the immune repertoire of the host it currently infects. Infection length was determined by the number of novel antigens the infecting strain presents to the host, with no explicit assumptions about expression order and no intrinsic differences between the two phenotypic groups. This might be seen in slight deviation from hypothesized fitness differences between UpsA and UpsB/C *var* genes, for example, where the former is often observed to dominate infections in young and immunologically naive hosts. However, by assuming an equal contribution by all variants, independent of phenotype, we create a more robust null-model, which does not rely on specific gene expression orders or growth rate differences in differently pre-exposed hosts.

With regards to immune interactions, we considered three different scenarios (i) variant-specific immunity only, (ii) phenotype-specific cross-immunity, and (iii) phenotype-transcending cross-immunity.

### 2.1. No cross-immunity

In the absence of any immune interactions, i.e. where immunity is solely variant-specific, we can define the length of infection, *L_n_*, simply by a linear function of the number of novel antigens of type *A* and *B* (*n*_A_ and *n*_B_, respectively), which in its simplest form could just be given as2.3
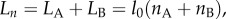
with *l*_0_ being the contribution of each antigen to infection length. As mentioned above, we did not assume any intrinsic difference between genes or phenotypes with regards to growth rate or immunogenicity, so each variant gene contributes equally to total infection length.

### 2.2. Phenotype-specific cross-immunity

We argued that antigens belonging to the same phenotype will have higher sequence similarities, and hence antigenic similarities, than antigens binding to different host receptors. We therefore considered the case whereby phenotype-specific cross-reactive immune responses would build up during the infection, which, in turn, would reduce the contribution of each additionally expressed variant—*of the same phenotype*—to infection length. The total infection length is then governed by a law of diminishing returns, given as2.4
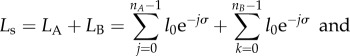
2.5
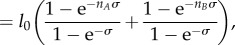
with *σ* being the degree of cross-reactivity (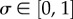). For most of this work, we assumed cross-immunity to be transient and reset to zero between consecutive infections; this was later relaxed, however, without changing the qualitative nature of our results.

### 2.3. Phenotype-transcending cross-immunity

Finally, we also considered the case where cross-immunity would build up during the infection in relation to any antigen presented, i.e. independent of their phenotypic group. In this case, the total infection length can be calculated as2.6
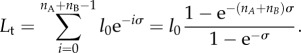


In order to investigate the robustness of evolved repertoire structures under different contributions of phenotype-specific and phenotype-transcending cross-immunity, we defined2.7
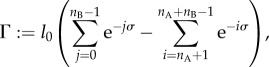


such that the infection length under the consideration of both phenotype-specific and phenotype-transcending cross-immunity becomes2.8
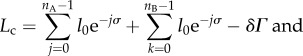
2.9
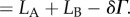
Therefore, *δ* = 0 corresponds to phenotype-specific cross-immunity only and *δ* = 1 corresponds to phenotype-transcending cross-immunity. Equally, setting *σ* = 0 recovers the infection length considering no cross-immunity at all.

## 3. Results

### 3.1. Variant-specific immunity selects against phenotypic diversity

**Figure 1.**
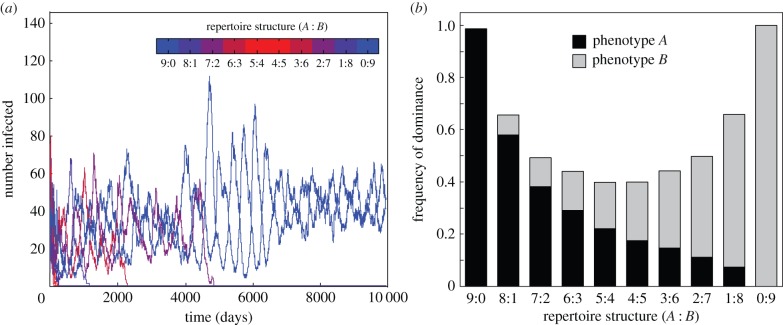
Default model behaviour under variant-specific immunity only. (*a*) Time series of a single model run show the changing prevalence of strains with differently structured repertoires, with strains coloured according to their repertoire structure (*A* : *B*). Phenotypically diverse strains are driven to extinction as a pair of strains consisting of only one or the other phenotype (i.e. with repertoire structures (9 : 0) and (0 : 9)) start to dominate. (*b*) Bar graph of the frequency by which different repertoire structures dominate the population, based on 10 000 model runs, shows how phenotypically less diverse parasites are favoured under variant-specific immunity. Stacked bars are used to visually represent within-repertoire phenotype distribution. Other parameter values: *r* = 9, *N*_A_ = 13, *N*_B_ = 13.

We first considered the case where host immunity is solely variant-specific, such that infection length is linearly correlated with the number of novel variants a parasite strain presents to its host (see Methods). Previous theory predicts that immune selection can cause parasite populations to organize into sets of dominant strains with minimal antigenic similarity. In this case, the immune responses induced by these strains cover a sufficiently large part of the available antigenic diversity to suppress the emergence or invasion of other (recombinant) strains [20,23]. We find a similar behaviour in our model system, where pairs of strains consisting of two homogeneous repertoires containing only one or the other phenotype, i.e. with a (phenotype *A* : phenotype *B*) repertoire structure of (*r* : 0) and (0 : *r*), which by definition share no antigen variants, more often end up being dominant with all other strains competitively excluded. Note that we consider a strain to be dominant if it is not extinct by *t* = 10 000. An example time series of this scenario is shown in figure 1*a*, where strains with phenotypically heterogeneous repertoires are driven to extinction and a set of strains with repertoire structures (*r* : 0) and (0 : *r*), and hence no antigenic overlap, become dominant.

Although the particular outcome of each simulation with respect to the repertoire structure that ends up dominating the population is highly stochastic and strongly dependent on the initial composition of strains, we find that on average strains whose repertoires are more biased towards one phenotype or another have a significant competitive advantage, simply because the chance of antigenic overlap between their respective repertoires is minimal compared with strains with a more even distribution of phenotypes within their repertoire. In other words, as strains become more balanced in terms of their antigens belonging to one or the other phenotypic group, the chance of overlap between them increases. This, on the other hand, reduces their population-level fitness as infection by one strain is more likely to induce partial immunity to another. This is shown in figure 1*b*, where we used average dominance frequency as an estimate of the (population-level) fitness of a particular repertoire structure. As mentioned in Methods, dominance is a qualitative measure referring to a strain or set of strains that is maintained in the population and has competitively excluded all other strains, as exemplified in figure 1*a*.

### 3.2. Phenotype-specific cross-immunity selects for partitioned gene repertoires

An important part of immune protection in malaria is the ability of anti-PfEMP1 antibodies to inhibit and potentially reverse binding of the infected red cells to host receptors [24,25]. It is therefore conceivable that antibodies raised against a variant of one phenotype should exhibit some cross-inhibition of binding of other variants of the same phenotype, but not necessarily inhibit the binding of variants that target a different host receptor. We therefore examined the effects of phenotype-specific cross-immunity on *var* gene repertoire evolution by further assuming that cross-immunity accumulates over the course of infection with each expressed variant of the same type. This, in turn, reduces the contribution of every additionally expressed variant to infection length, resulting in the total infection length, *L_n_*, in relation to the number of novel antigens exposed to the host, being governed by a law of diminishing returns (see Methods for more details).

**Figure 2.**
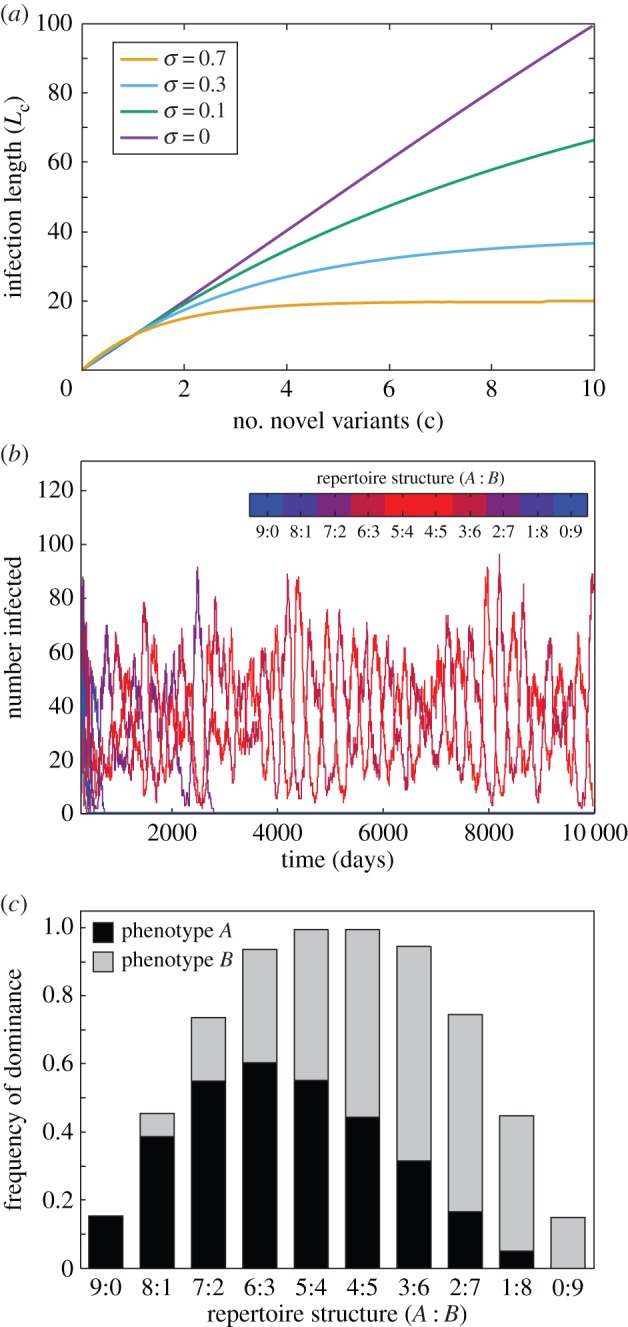
Cross-immunity selects for phenotypically diverse repertoires. (*a*) Total infection length as a function of the number of antigenic variants presented over the course of infection, *c*, under variation in the degree of cross-immunity, *σ*. For high levels of cross-immunity, infection length starts to plateau well before the parasite can exhaust its antigenic repertoire and additionally expressed genes will not contribute to further infection length. (*b*) Time series of a single model run with temporary cross-immunity (*σ* = 0.3) showing the competitive exclusion of phenotypically uniform parasites (blue lines) in favour of strains containing partitioned and hence phenotypically diverse repertoires (red and purple lines). (*c*) Bar graph of the average strain dominance frequency based on 10 000 model runs shows how phenotypically diverse strains gain a selective advantage under phenotype-specific cross-immunity (*σ* = 0.3). Stacked bars are used to visually represent within-repertoire phenotype distribution. Other parameter values: *r* = 9, *N*_A_ = 13, *N*_B_ = 13.

Depending on the degree of cross-immunity, *σ*, we note that a strain's infection length, even in naive hosts, quickly plateaus with the number of expressed variants, as shown in figure 2*a*, implying there is an upper limit to the length of an infection which cannot notably be extended by expressing additional variants of the same phenotype. Crucially, however, this limitation can be circumvented by the parasite by expressing members of an alternative phenotypic group.

Under the addition of phenotype-specific cross-immunity, antigen repertoires now experience selection pressure by two conflicting forces. At the population level, the pressure to reduce the probability of encountering hosts with prior immunity selects for homogeneous repertoire structures and low phenotypic diversity, which minimizes antigenic overlap with other strains (as shown above, figure 1). At the level of individual infections, on the other hand, repertoires are under selection pressure to maximize infection length, which is best satisfied by having phenotypically diverse repertoires containing equal numbers of each phenotypic group. As a result, under moderate-to-high degrees of cross-immunity, *σ*, we now find phenotypically diverse strains dominating the population, as shown by an example time series and repertoire structure dominance frequency in figure 2*b* and *c*, respectively.

**Figure 3.**
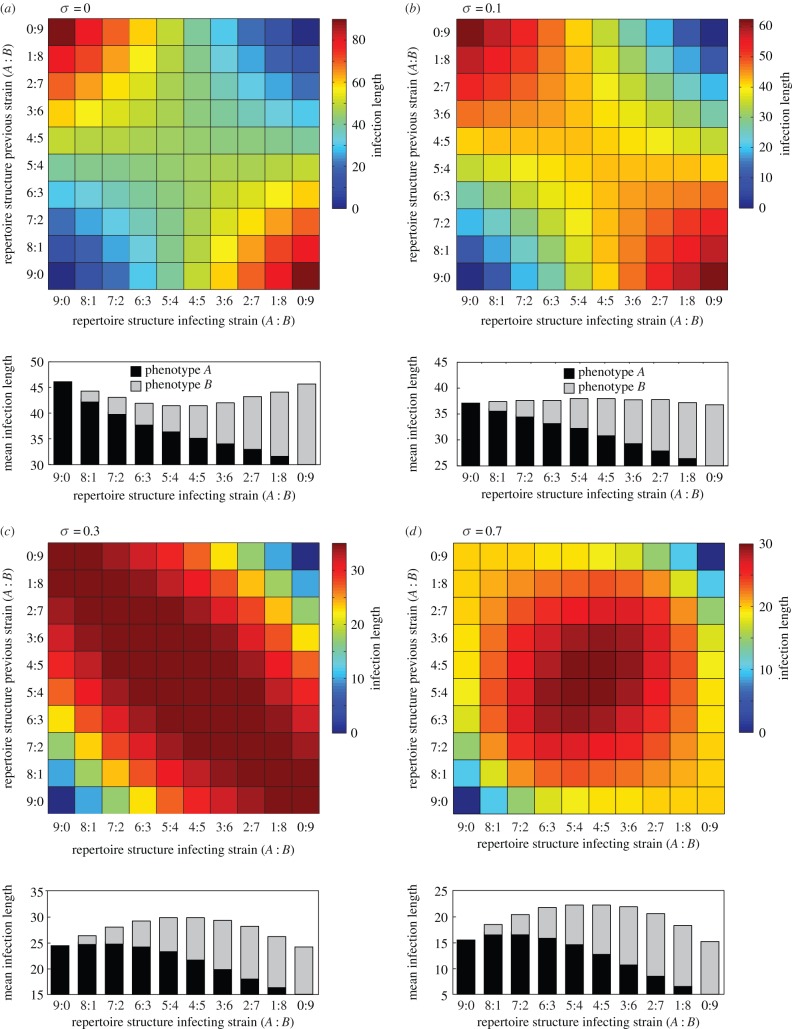
Pairwise and mean infection length under changes in cross-immunity. (*a–d*) Matrix plots show the (*i*,*j*) pairwise infection lengths, resulting from an infection of strain *i* in a host previously exposed to strain *j*. The bar graphs depict the mean infection length of each repertoire structure (represented as stacked bars to represent within-repertoire phenotype distribution), derived by averaging the strains' length of infection against all non-self-strains (i.e. the column mean of the pairwise infection matrix). As cross-immunity is increased ((*a–d*)), phenotypically diverse repertoires start to gain a competitive advantage. Note that different scales are used to illustrate the qualitative change in repertoire fitness. (*a*) *σ* = 0, (*b*) *σ* = 0.1, (*c*) *σ* = 0.3, (*d*) *σ* = 0.7. Other parameter values: *r* = 9, *N*_A_ = 9, *N*_B_ = 9.

To further understand and illustrate the evolution of different repertoire structures under changes in the degree of cross-immunity, we can calculate the relative fitness of a particular strain by means of its infection length in a host previously infected by any of the other strains. For illustration purposes only, we restrict the analysis to a limited antigenic pool where the total number of variants of phenotype *A* or *B* equals the parasite's repertoire size, such that a pair of phenotypically homogeneous strains would completely exhaust the available antigenic space. The matrix plots in figure 3 show the infection length of strain *i* in a host with previous exposure to strain *j* for all (*i*,*j*)-pairs, which clearly demonstrate how phenotypically diverse repertoires start to gain a competitive advantage as the degree of cross-immunity increases (from *σ* = 0, (*a*), to *σ* = 0.7, (*d*)), leading to their preferred selection and dominance within the population.

Importantly, the selection of phenotypically diverse strains is robust to parameter changes in host population sizes, repertoire size, force of infection and antigen expression order and further persists, regardless of whether we assume cross-reactive immunity to be transient and lost between infections or whether it is permanent and accumulates over repeated infections (shown in electronic supplementary material, figures S1–S5). We also tested the robustness of these results by relaxing our assumption of cross-immunity to be restricted to the respective phenotypes (see Methods). We now find that as cross-immunity shifts away from being confined to variants of the same phenotype to become more predominantly phenotype transcending, parasite repertoires become phenotypically less diverse. As shown in electronic supplementary material, figure S4, even moderate-to-high degrees of phenotype-transcending immunity can select for within-repertoire phenotypic diversity as long as there is a non-negligible degree of phenotype-specific cross-immunity.

### 3.3. Functional constraints influence repertoire partitioning

Data from fully sequenced *var* gene repertoires reveal a conserved structure with very uneven group sizes. We therefore investigated possible conditions that could offer an evolutionary advantage of skewed phenotype distributions, concentrating again on the functionality of PfEMP1 in terms of receptor binding.

It can be argued that the evolution and diversification of PfEMP1 variants is constrained by the necessity to retain functional binding to their target receptors. It is further possible that different binding phenotypes, which target different host receptors, are constrained to different degrees, for example, owing to the specific structural characteristics of the binding targets. On the one hand, this may limit the global antigenic diversity of each phenotype to a different degree, i.e. resulting in different global pool sizes *N*_A_ and *N*_B_. On the other hand, it may result in different degrees of cross-reactive inhibition experienced by variants of each phenotype, i.e. where the degree of cross-immunity, *σ*, becomes phenotype-specific (*σ*_A_ and *σ*_B_).

**Figure 4.**
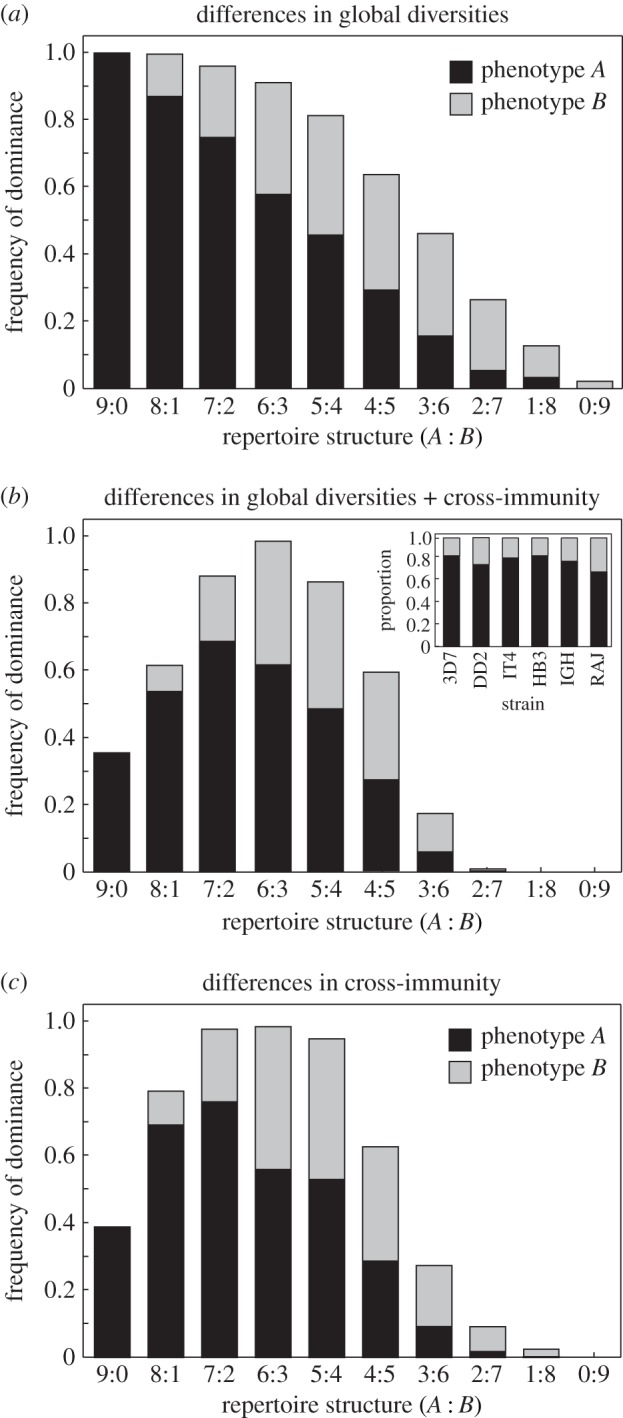
Functional constraints on PfEMP1 select for skewed phenotype distributions. Bar graphs show the average strain dominance frequencies based on 10 000 model runs, with stacked bars used to represent within-repertoire phenotype distribution. (*a*) Considering different antigenic pool sizes of phenotypes *A* and *B* (*N*_A_ = 36 and *N*_B_ = 9) with variant-specific immunity only selects for parasites with mono-phenotypic repertoires. (*b*) Including cross-immunity (*σ* = 0.1) restricts the benefit of expressing more variants of the same phenotype, leading to the evolution of phenotypically diverse repertoires skewed towards the more diverse phenotype (*A*). The inset shows the repertoire distribution of UpsA versus non-UpsA *var* genes in seven sequenced *P. falciparum* isolates, shows a skewed and conserved repertoire partitioning. (*c*) Differences in the degree of cross-reactivity, with *σ*_A_ = 0.1 and *σ*_B_ = 0.8, has the same effect as different antigenic pool sizes and select for repertoires with uneven numbers of phenotypic variants. Other parameter values: *r* = 9.

We first examined the influence of global antigen diversity on the repertoire partitioning structure by considering differently sized antigen pools of the two phenotypic groups *a* and *b*. We note that by increasing the size of one phenotype's antigen pool, the likelihood of overlaps within a particular phenotype group decreases. In the absence of cross-immunity, this results in a competitive advantage to repertoire structures that use larger numbers of this (more diverse) phenotype (figure 4*a*). With the addition of cross-reactive immunity, the extent to which large numbers of antigens from this phenotype group can be usefully exploited is curtailed, however. This results in a skewed distribution in repertoire structure dominance frequency, where homogeneous repertoire structures (with low phenotypic diversity) are strongly disadvantaged while moderately heterogeneous repertoires with a bias towards the more diverse phenotype group are favoured (figure 4*b*).

Next, we considered differential degrees by which antibodies can inhibit PfEMP1 binding in a phenotype-specific manner. We argued that because PfEMP1 is a modular protein, distinct variants may still share whole domains or exhibit regions of high similarity between domains. Furthermore, domains can be organized into motifs comprised of specific domain subclasses in tandem that are commonly referred to as domain cassettes. The above-mentioned functional constrains on domain diversity together with observed correlations between groups of domain subclasses, which may act as functional units, could limit the number of antigenically distinct binding epitopes and thus increase the degree of cross-reactivity within variants containing similar domains. Broad inhibitory cross-reactivity at the domain/domain cassette level has been shown previously [25] and while we do not model the domain-level structure of PfEMP1 explicitly, we incorporated differences in cross-reactivity by using phenotype-specific values of *σ*. The results are similar to those obtained by considering different antigenic pool sizes, leading to skewed phenotype distributions whereby strains with moderately heterogeneous repertoires can be seen to enjoy an evolutionary advantage, resulting in frequent selection and dominance in the population (figure 4*c*), in line with empirical observations (inset in figure 4*b*).

## 4. Discussion

The highly immunogenic nature of the *var* gene-encoded malaria virulence factors PfEMP1 makes them important targets for host protective immune responses and therefore subject to intense selection pressure. Previous theoretical work on the evolution of *var* gene repertoires has predicted that strong immune selection will act to structure the parasite population into strains with minimal antigenic similarity [20], such that an infection by one strain has little or no detrimental impact on the fitness of subsequently infecting strains. Since then, further empirical work on *var* genes and *var* gene repertoires has highlighted a conserved genomic structuring, whereby gene repertoires are not just random collections of antigenic variants, but contain distinct groups of genes differentiated for example by their genomic location, upstream promoter sequence, number of binding domains or even sequence diversity [6,17,22]. These characteristics are interrelated, however, and there appears to be some mechanism in place that maintains the observed repertoire structure over evolutionary time [26] despite high rates of recombination. This, on the other hand, strongly suggests that gene repertoires containing distinct groups of genes have a competitive advantage despite the poor utilization of the globally antigenic diversity this entails [21], especially when considering that the ratios of genes belonging to the different groups also appear highly conserved [6,16,17].

In line with previous predictions, we found that variant-specific immune responses can select for parasite strains with minimally overlapping antigenic repertoires. Considering that antigenic variants can be of two different phenotypes, this implied an evolutionary trend towards the dominance of pairs of strains comprising variants of only one or the other phenotype. We note that this particular outcome was partly driven by our model set-up to contain one representative strain of every possible repertoire structure. In this case, two strains whose repertoires contained variants of only one or the other phenotypic group will be (antigenically) non-overlapping by default, whereas the probability that two strains containing variants of both phenotypic groups have overlapping repertoires increases as the phenotype distribution becomes more even. On the other extreme, when we initialized the model such that each strain has a non-overlapping counterpart, there are no longer population-level fitness differences and all strains will be equally likely to become dominant. One way or the other, this clearly illustrates that variant-specific immunity alone cannot account for the empirically observed phenotypic diversity within *var* gene repertoires.

We therefore concentrated on the functional importance of PfEMP1, i.e. adhesion to host cell receptors, as a potential factor in *P. falciparum* evolution. Because of its crucial contribution to within-host parasite fitness, by removing the infected red cells from circulation and thus avoiding clearance by the spleen, we argued that (antigenic) diversification of PfEMP1 may be limited for the protein to retain functional viability, and we further argued that this limitation would increase the chance of antibody-mediated cross-inhibition of parasite binding. The presence of transient cross-reactive immune responses has been well established in longitudinal studies [27,28], although whether these are specific to particular phenotypes or not is not yet clear. More recent work has confirmed the presence of cross-reactive immunity between variants with specific binding phenotypes, for example in IgM rossetting [29], as well as broad cross-reactivity to particular epitope regions, such as domain cassette 4, which is associated with ICAM binding [25], where both binding phenotypes are associated with severe infection pathology. Furthermore, structural conservation in EPCR-binding PfEMP1 has been shown despite large sequence diversity [30], although the contribution this makes to cross-reactive antibody-mediated immunity, especially over the course of an infection, is yet to be established.

We tested two PfEMP1-binding related hypotheses for their effect on the evolution of intragenomic repertoire partitioning: (i) phenotype-specific differences in the degree by which the proteins, or particular binding loci, can diversify without losing functionality, leading to different pool sizes of antigenic variants and (ii) phenotype-specific differences in the degree of cross-inhibition. Both could, in fact, be argued to be part of the same process, such that a constraint on protein diversification might entail an increase in cross-recognition by antibodies. In terms of their role in gene repertoire evolution, we found that both had the same effect and caused a shift in repertoire structuring towards biased phenotype distributions, similar to those observed in *P. falciparum*. Based on features of short sequence tags within the Duffy-binding-like *α* domain, Bull *et al.* [31] reported similar frequency distributions of groups of *var* genes within the genome as within the population, despite them being very different in size. Similarly, it is well known that the UpsA group of *var* genes exhibits less global diversity than other groups and also comprises a relatively small proportion of the *var* gene repertoire. The most extreme example might be that of *var*2*csa*, whose presence in every repertoire and unusual sequence conservation compared with other *var* genes makes it an attractive vaccine target [32]. Further empirical investigations are necessary to test whether these observations could, in fact, be due to our hypothesized functional constraints or differences in immunogenic cross-inhibition, however.

We assumed that variant genes are being expressed sequentially over the course of an infection, at least to a level sufficiently high to trigger an immune response. Under this assumption, each additionally expressed gene contributes positively to infection length and thus transmission probability. In the most extreme case, this led to every variant having an equal contribution to the length of infection and thus within-host fitness, which is highly unlikely to be the case in real malaria infection. On the other hand, this particular scenario could not explain the observed repertoire structures. Once we considered cross-immunity, this assumption was partially relaxed and we showed that as long as individual variants would contribute positively to within-host fitness, i.e. as long as infection length was a monotonically increasing function of antigens expressed during infection, the model robustly predicted evolution towards phenotypically diverse repertoire structures. Furthermore, it is also worth mentioning that the sequential dominance by a single or only a few antigenic variants is thought to underlie the characteristic waves of parasitaemia in *P. falciparum* malaria [33–35]. In that respect, it is also interesting to note that transient cross-reactive immune responses against groups of antigenic variants sharing particular PfEMP1 epitopes have previously been proposed to orchestrate the parasite population to exhibit the observed single-variant dominance during infection [36]. This adds further to the notion that cross-reactive immunity could play a highly significant role for parasite evolution and malaria epidemiology in general.

Another interesting consequence of transient cross-reactive immunity is that it offers a simple explanation as to why *var* gene repertoires have evolved to be limited to ≈60 variants. Under variant-specific immunity alone, we would expect either selection for vast repertoires of antigenically distinct variants or the evolution of a mechanism by which diversity can easily be generated during the course of infection, both of which are common strategies for other antigenically variable organisms [37]. Although high rates of mitotic recombination in culture adapted *P. falciparum* isolates have recently been reported [38], it is not yet clear if and to what degree this could contribute to immune evasion during infection or whether its role is more limited towards the generation of antigenic diversity at the population level. Therefore, the relatively limited size of the antigenic repertoire of *P. falciparum* has been a bit of a conundrum, which temporary cross-immunity could possibly resolve, although this is purely speculative at this stage and requires further investigation.

In summary, our results support the hypothesis that phenotypically diverse *var* gene repertories are maintained by a combination of between-strain and within-host immune selection. A similar mechanism has recently been put forward to underlie the sequence and domain evolution of individual *var* genes and gene repertoires [22], which strongly suggests that evolutionary trade-offs, by which the parasite has to optimize between the within-host and within-population-level fitness, are important determinants in *P. falciparum* evolution.

## Supplementary Material

Supplementary figure legends

## Supplementary Material

Figure S1

## Supplementary Material

Figure S2

## Supplementary Material

Figure S3

## Supplementary Material

Figure S4

## Supplementary Material

S5 global crossreactivity.pdf
