# Supplementary material for: Maintenance of phenotypic diversity within a set of virulence encoding genes of the malaria parasite Plasmodium falciparum
Source: J R Soc Interface. 2015 Dec 6;12(113):20150848. doi: 10.1098/rsif.2015.0848 (PMC4707858; doi:10.1098/rsif.2015.0848)
Supplement: Supplementary figure legends [file rsif20150848supp1.pdf]

Supplementary Figure Legends

Figure S1. Dominance frequency of repertoire structures under changes to repertoire variant pool sizes. The distribution of dominant repertoire structures under phenotype-specific cross-immunity ($\sigma = 0.3$) favours phenotypically diverse repertoires and is robust to repertoire size (r) and global antigen pool sizes (N_A, N_B). (a) $r = 5$, $N_A = 13$, $N_B = 13$, (b) $r = 21$, $N_A = 21$, $N_B = 21$. and (c) $r = 61$, $N_A = 61$, $N_B = 61$. Dominance frequency based on 4000 model runs.

Figure S2. Dominance frequency of repertoire structures under changes to transmission intensity. Average dominance frequency for increasing infection rates (λ_0=0.0033 (a), λ_0=0.033 (b) and λ_0=0.066 (c)) shows how selection for phenotypically diverse repertoires under phenotype-specific cross-immunity () is unaffected by the force of infection. . Other parameter values: $r = 9$, $N_A = 13$, $N_B = 13$, $\sigma = 0.3$. Dominance frequency based on 10,000 model runs.

Figure S3. Evolved repertoire structures for random and *non-random* expression order. The distribution of dominant repertoire structures is robust to the order of antigen expression. (a) Expression order is randomised before each infection. (b) Antigen expression is ordered by phenotype group such that all *A* phenotype antigens are expressed first. In both cases $\sigma = 0.3$. Other parameter values: r = 9, $N_A = 13$, $N_B = 13$. Dominance frequency calculated based on 5,000 model runs.

Figure S4. Evolved repertoire structures for transient and permanent cross-immunity. The distribution of dominant repertoire structures is robust to assumption about duration of cross-immunity, i.e. whether it is (a) transient and reset between infections, or (b) permanent and builds up over the host's lifetime. In both cases $\sigma = 0.3$. Other parameter values: $r = 9$, $N_A = 13$, $N_B = 13$. Dominance frequency calculated based on 5,000 model runs.

Figure S5. Dominance frequency of repertoire structures for phenotype-specific and phenotype-transcending immunity. The distribution of dominant repertoire structures under cross-reactive immunity ($\sigma = 0.3$) for different δ, where δ specifies a balance between phenotype-specific ($\delta = 0$) and phenotype-transcending ($\delta = 1$) immunity. Phenotypically diverse repertoire structures are selected for under low to moderate δ (a), but break down as phenotype-transcending immunity becomes very strong (b) and (c). (a) $\delta = 0.6$, (b) $\delta = 0.8$, (b) $\delta = 0.9$. Other parameter values: $r = 9$, $N_A = 13$, $N_B = 13$. Dominance frequency calculated based on 10,000 model runs.
