# Supplementary figures and images for: Maintenance of phenotypic diversity within a set of virulence encoding genes of the malaria parasite Plasmodium falciparum
Source: J R Soc Interface. 2015 Dec 6;12(113):20150848. doi: 10.1098/rsif.2015.0848 (PMC4707858; doi:10.1098/rsif.2015.0848)

(a)

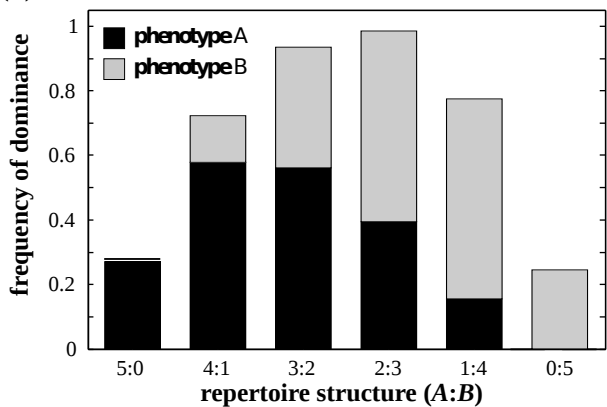

(b)

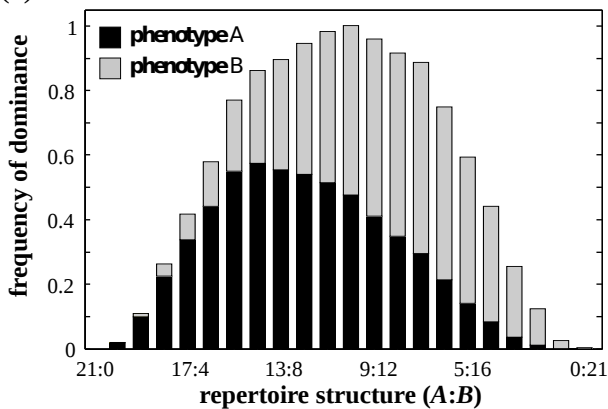

(c)

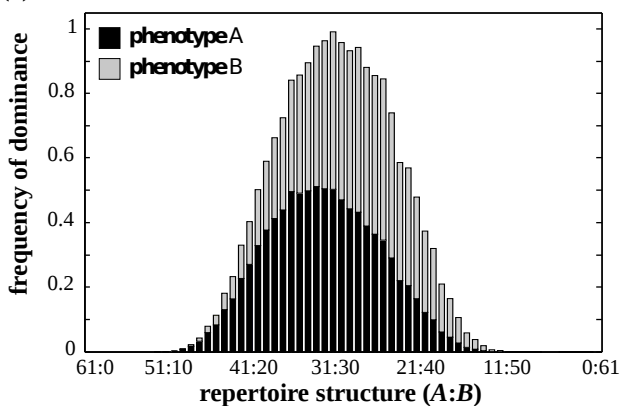

Supplement: Figure S1 [file rsif20150848supp2.pdf]

(a)

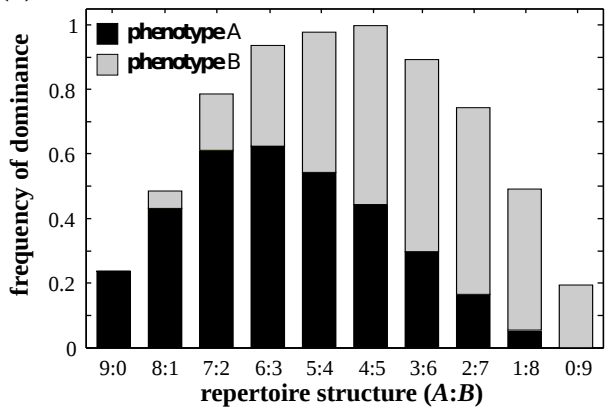

(b)

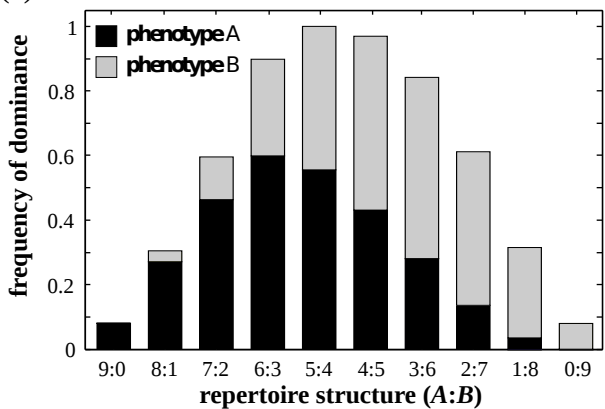

(c)

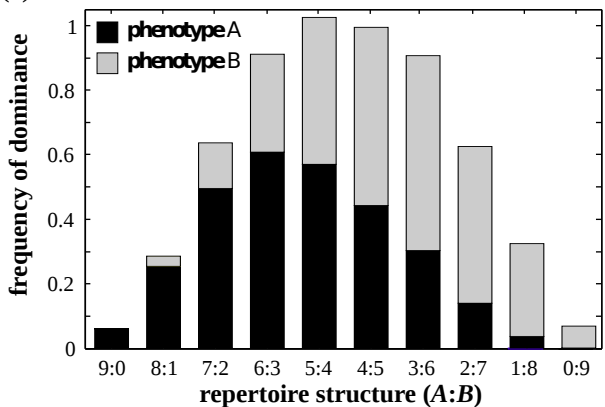

Supplement: Figure S2 [file rsif20150848supp3.pdf]

randomised expression order

(a)

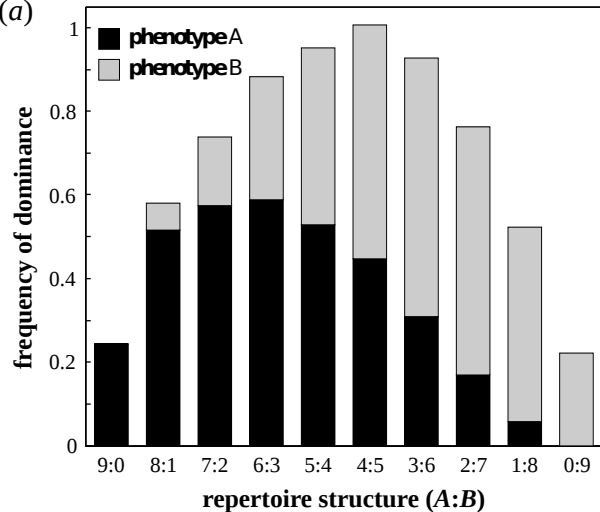

A-first expression order

(b)

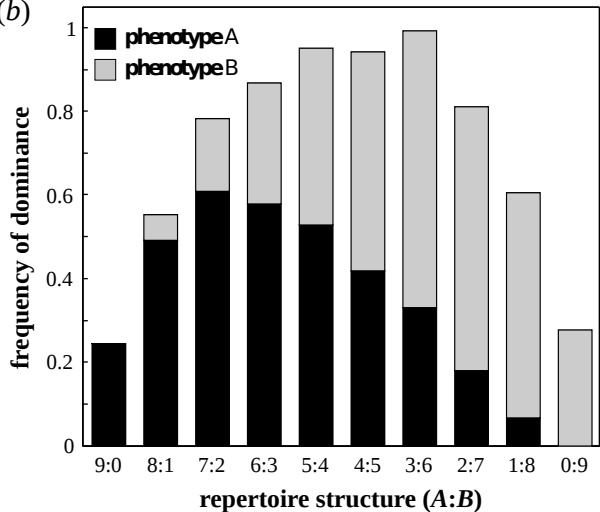

Supplement: Figure S3 [file rsif20150848supp4.pdf]

transient crossreactivity

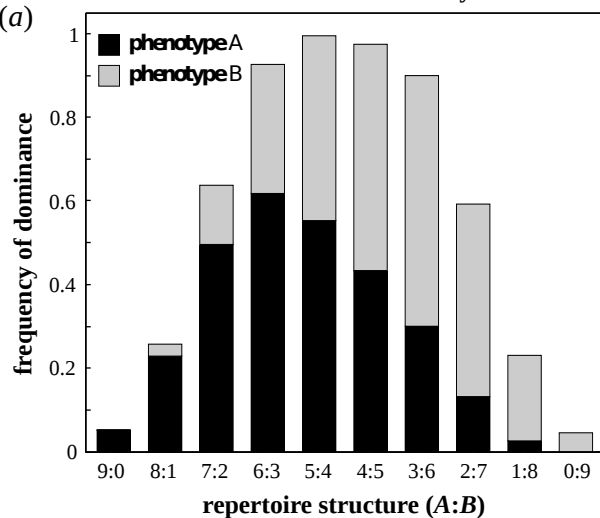

permanent crossreactivity

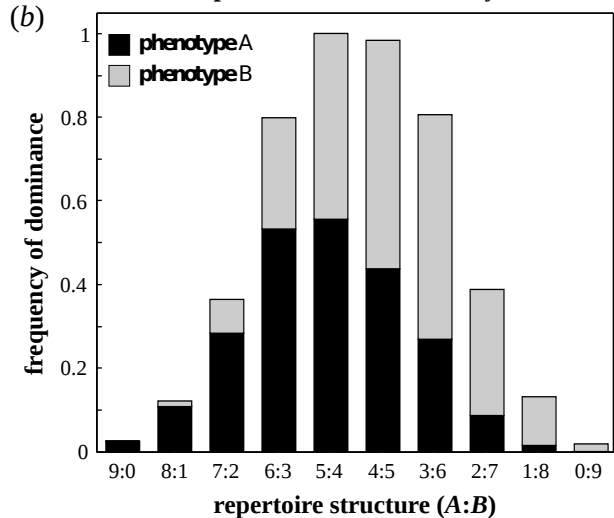

Supplement: Figure S4 [file rsif20150848supp5.pdf]

(a)

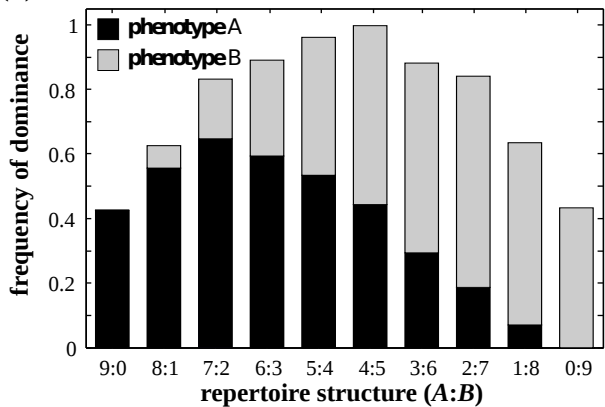

(b)

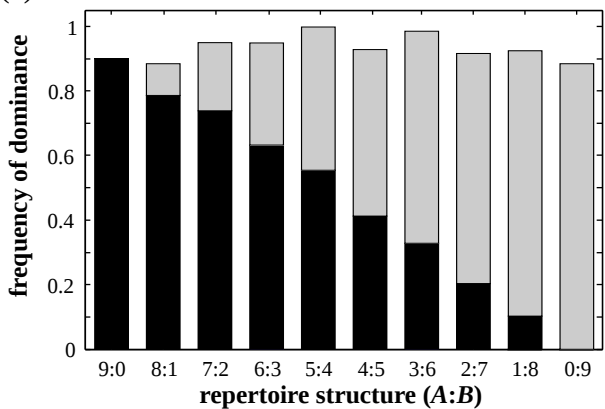

(c)

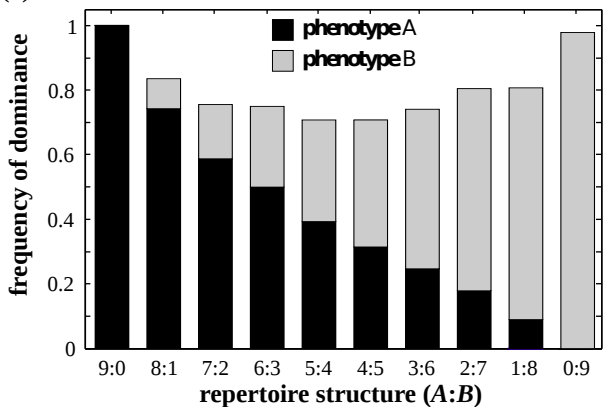

Supplement: S5 global crossreactivity.pdf [file rsif20150848supp6.pdf]
